# Genotype-Specific HPV E6/E7 mRNA Triage for Risk Stratification in HPV DNA-Positive Women with ASC-US/LSIL: A Population-Based Tromsø Cohort

**DOI:** 10.3390/pathogens15070747

**Published:** 2026-07-17

**Authors:** Sveinung Wergeland Sørbye, Bente Marie Falang, Mona Antonsen, Elin Richardsen

**Affiliations:** 1 Department of Clinical Pathology, University Hospital of North Norway, 9006 Tromsø, Norway; mona.antonsen@unn.no (M.A.); elin.richardsen@unn.no (E.R.); 2 PreTect AS, 3490 Asker, Norway; bente.falang@pretect.no

**Keywords:** HPV DNA, HPV E6/E7 mRNA, PreTect HPV-Proofer’7, ASC-US, LSIL, CIN2+, CIN3+, molecular triage, extended genotyping, colposcopy referral, cervical cancer screening

## Abstract

HPV DNA-positive women with ASC-US/LSIL cytology constitute a heterogeneous triage group. We evaluated genotype-specific HPV E6/E7 mRNA testing with PreTect HPV-Proofer’7 in a population-based cohort from Tromsø and compared the findings descriptively with a previously published cohort from Bodø. This retrospective quality-assurance study included 1006 HPV DNA-positive women with ASC-US/LSIL cytology screened between 2019 and 2024, with linkage to regional pathology records through 31 December 2025. The assay detects E6/E7 mRNA from HPV16, 18, 31, 33, 45, 52, and 58. ASC-US/LSIL accounted for 42.5% of linked HPV DNA-positive women in Tromsø compared with 22.3% in Bodø. Among the mRNA-tested ASC-US/LSIL cohorts, mRNA positivity was similar (40.8% vs. 44.6%). In Tromsø, CIN2+ was recorded in 26.1% of mRNA-positive and 7.9% of mRNA-negative women (RR 3.31, 95% CI 2.41–4.55), whereas CIN3+ was recorded in 3.9% and 1.0%, respectively (RR 3.88, 95% CI 1.53–9.82). For CIN2+, sensitivity was 69.5%, specificity 64.4%, PPV 26.1%, and NPV 92.1%. Among HPV16/18 DNA-positive women, CIN2+ risk was 42.7% in mRNA-positive and 13.4% in mRNA-negative women. Simulated mRNA-guided referral reduced immediate colposcopy referrals by 59.2%. Genotype-specific mRNA testing provided clinically relevant risk stratification, with directionally similar patterns being observed in the Tromsø and Bodø cohorts; however, mRNA-negative women require structured surveillance.

## 1. Introduction

Primary human papillomavirus (HPV) DNA screening has increased the sensitivity of cervical cancer prevention programmes, but its limited specificity creates a need for effective triage of HPV-positive women [1,2,3]. Current triage approaches include reflex cytology, partial or extended HPV genotyping, p16/Ki-67 dual-stain cytology, and other molecular biomarkers, with the optimal strategy depending on diagnostic performance, available resources, and the capacity for structured follow-up [4,5,6,7,8]. Reflex cytology remains widely used because it provides morphological information and can identify high-grade abnormalities requiring prompt evaluation [8,9]. However, cytological interpretation is observer-dependent, particularly for atypical squamous cells of undetermined significance (ASC-US) and low-grade squamous intraepithelial lesions (LSIL) [10,11,12,13]. These categories comprise a heterogeneous group: some women have underlying CIN2+ or CIN3+, whereas others have transient HPV-associated changes, inflammation, atrophy, or lesions with a high probability of regression [9,11,12,14]. Interlaboratory variation in the threshold for assigning ASC-US or LSIL may therefore influence both the proportion of HPV DNA-positive women entering the low-grade cytology pathway and the average disease risk within that subgroup [10,13,15]. More objective molecular triage markers may help refine risk and reduce unnecessary colposcopy referrals while maintaining appropriate surveillance for women at lower immediate risk [3,16,17]. Molecular triage may become increasingly relevant as HPV self-sampling expands, because cytology-dependent pathways may require recall for collection of a cervical specimen by a clinician [4,18,19,20].

HPV E6/E7 messenger ribonucleic acid (mRNA) testing provides biological information that differs from HPV DNA detection. Whereas HPV DNA assays identify the presence and genotype of viral DNA, E6/E7 mRNA detection provides evidence of viral oncogene transcription, which is more closely associated with HPV-driven cellular transformation [21,22]. PreTect HPV-Proofer’7 is a genotype-specific assay that detects E6/E7 mRNA from HPV16, 18, 31, 33, 45, 52, and 58 [23]. These genotypes are among those most frequently associated with cervical high-grade lesions and cancer and correspond to the oncogenic HPV types included in the nonavalent HPV vaccine [24,25]. Genotype-specific mRNA testing may therefore combine type-specific risk information with evidence of transcriptional activity. Previous studies have shown that HPV E6/E7 mRNA testing can reduce test positivity and improve specificity compared with broader HPV DNA-based strategies, although performance varies by assay configuration, genotype coverage, population, and clinical setting [22,23,26,27,28].

A recent real-world cohort from Bodø evaluated the same 7-type HPV E6/E7 mRNA assay in 175 HPV DNA-positive women with ASC-US/LSIL cytology and reported risk stratification by mRNA status together with fewer simulated immediate colposcopy referrals [29]. However, the modest cohort size limited precision, particularly for CIN3+ and genotype-specific estimates. Evaluation in a larger population-based cohort is therefore needed to determine whether mRNA status continues to distinguish women with different recorded risks of high-grade disease within this clinically heterogeneous subgroup. The Tromsø cohort also provides an opportunity to examine an additional scientific question: whether marked differences in the proportion of HPV DNA-positive women classified as ASC-US/LSIL across two pathology laboratories are accompanied by similar or different patterns of molecular triage positivity and risk stratification. Because the Tromsø and Bodø cohorts differed in study period, size, screening implementation, and follow-up, this comparison was considered descriptive and was not intended as formal external validation.

We hypothesised that detection of E6/E7 mRNA from one or more of the seven targeted HPV genotypes would identify a subgroup of HPV DNA-positive women with ASC-US/LSIL cytology who had a higher recorded risk of CIN2+ and CIN3+ than mRNA-negative women. The primary objective was to evaluate CIN2+ risk stratification and diagnostic performance of 7-type HPV E6/E7 mRNA testing in a population-based Tromsø cohort screened between 2019 and 2024, with linkage to regional pathology records through 31 December 2025. CIN3+ was evaluated as a secondary endpoint. Supportive analyses assessed genotype-specific risks, HPV16/18 DNA–mRNA discordance and CIN2+ risk within the HPV16/18 DNA-positive subgroup, and the potential reduction in immediate colposcopy referrals under a deterministic mRNA-guided referral scenario. Selected findings were also compared descriptively with the previously published Bodø cohort to explore how differences in low-grade cytology classification might influence the composition and risk profile of the triage population [29].

## 2. Materials and Methods

### 2.1. Study Design, Setting, and Population

This single-centre retrospective quality-assurance cohort study was conducted at the Department of Clinical Pathology, University Hospital of North Norway, Tromsø, Norway. The study was embedded in routine primary HPV DNA screening within the Norwegian Cervical Cancer Screening Programme and included women screened between 1 January 2019 and 31 December 2024. During 2019–2022, primary HPV DNA screening was offered to women aged 34–69 years; during 2023–2024, the programme was expanded to include women aged 25–69 years. Regional pathology records, including cytology, HPV testing, cervical histology, and treatment specimens, were available through 31 December 2025. Histological verification was performed according to routine clinical management and was not systematic for all women.

During the study period, 42,791 women underwent primary HPV DNA screening at the University Hospital of North Norway. HPV DNA was detected in 2405 women (5.6%). Of these, 37 were excluded from the linked HPV DNA-positive cohort: 31 because of insufficient residual sample volume, four because of an invalid 7-type HPV mRNA result, and two because a Norwegian personal identification number was unavailable for reliable linkage. The linked HPV DNA-positive cohort therefore comprised 2368 women.

Among these 2368 women, 1362 (57.5%) had reflex cytology other than ASC-US or LSIL and were not included in the present analysis. The final analytic cohort comprised 1006 HPV DNA-positive women with ASC-US/LSIL cytology, including 659 women with ASC-US (65.5%) and 347 women with LSIL (34.5%). All included women had a valid 7-type HPV mRNA result and linkage to regional pathology records through 31 December 2025.

### 2.2. Data Sources, Linkage, and Quality Control

All study data were obtained from routine laboratory records at the Department of Clinical Pathology, University Hospital of North Norway (UNN), using the laboratory information system SymPathy (Tietoevry, Espoo, Finland). UNN is the sole pathology provider for Troms and Finnmark, allowing for near-complete regional capture of cervical screening samples, HPV test results, cytology diagnoses, cervical biopsies, and treatment specimens. The dataset included primary HPV DNA screening results, reflex cytology diagnoses, 7-type HPV mRNA results, and follow-up pathology records through 31 December 2025.

Individual-level linkage between the baseline screening episode and subsequent follow-up records was performed using the Norwegian personal identification number. Women without a valid Norwegian personal identification number were excluded because reliable linkage to follow-up pathology records could not be ensured.

Quality control was performed before analysis. The dataset was checked for duplicate records, valid HPV DNA and mRNA results, consistency between sample identifiers and collection dates, completeness of cytology classification, and agreement between morphology codes and Bethesda categories. The final analytic dataset was reviewed to confirm that all included women had a positive primary HPV DNA screening result, ASC-US or LSIL cytology, a valid 7-type HPV mRNA result, and successful linkage to regional pathology records. Implausible or inconsistent entries were checked against the original SymPathy records before analysis.

Histological follow-up status was classified according to whether relevant cervical histology was recorded on or after the index screening date through 31 December 2025. Histological follow-up included cervical biopsies, endocervical curettage specimens, conisation specimens, and other relevant treatment specimens. For women with histological follow-up, the most severe diagnosis recorded during the observation period was retained. For endpoint analyses, women without a recorded CIN2+ or CIN3+ diagnosis were classified as not having reached the respective endpoint during the available follow-up period; this classification should not be interpreted as histologically confirmed absence of disease. Cytology- and/or HPV-based follow-up without histology was not separately classified in the present data extraction and therefore could not be distinguished from absence of later registered testing.

### 2.3. Screening Tests and Laboratory Workflow

Primary HPV DNA screening was performed as part of routine cervical cancer screening at the Department of Clinical Pathology, UNN. Cervical samples were clinician-collected in PreservCyt^®^ medium (ThinPrep^®^, Hologic, Marlborough, MA, USA) and analysed using the cobas^®^ 4800 HPV Test (Roche Molecular Systems, Pleasanton, CA, USA) according to standard laboratory procedures. The cobas assay reports HPV16 and HPV18 separately and provides a pooled result for 12 additional oncogenic HPV types: 31, 33, 35, 39, 45, 51, 52, 56, 58, 59, 66, and 68.

For all HPV DNA-positive samples, reflex liquid-based cytology was performed using residual material from the same PreservCyt^®^ vial. Cytological diagnoses were reported according to Bethesda terminology.

Residual PreservCyt^®^ material from the original screening sample was also used for genotype-specific HPV E6/E7 mRNA testing with PreTect HPV-Proofer’7 (PreTect AS, Asker, Norway). The assay detects E6/E7 mRNA from HPV16, 18, 31, 33, 45, 52, and 58. Samples were classified as mRNA-positive when at least one of the seven genotype-specific mRNA targets was detected.

Because HPV DNA testing, reflex cytology, and HPV mRNA testing were performed on material from the same clinician-collected PreservCyt^®^ sample, the retrospective molecular triage assessment did not require repeat sampling. Clinical management during the study period followed routine screening practice and was based on HPV DNA and cytology results. The mRNA results did not influence colposcopy referral, biopsy practice, treatment decisions, or follow-up.

### 2.4. Cohort Definition, Outcome Definitions, and Genotype Classification

The analysis was restricted to the predefined ASC-US/LSIL subgroup within the linked HPV DNA-positive screening cohort. This subgroup was selected because low-grade cytology is common in HPV-based screening and represents a clinically heterogeneous triage category in which additional molecular risk stratification may be useful. The same cytology-defined subgroup was evaluated in the previously published Bodø cohort, allowing for descriptive cross-cohort comparison between two Norwegian laboratory settings.

The primary endpoint was CIN2+, defined as cervical intraepithelial neoplasia grade 2, cervical intraepithelial neoplasia grade 3, adenocarcinoma in situ, or invasive cervical carcinoma. CIN3+ was evaluated as a secondary endpoint and included cervical intraepithelial neoplasia grade 3, adenocarcinoma in situ, or invasive cervical carcinoma. Endpoint classification was based on cervical histopathology recorded in SymPathy through 31 December 2025. When more than one histological diagnosis was recorded for the same woman during follow-up, the most severe diagnosis was used. For the CIN2+ analysis, women without recorded CIN2+ during follow-up were classified as not having reached the CIN2+ endpoint. For the CIN3+ analysis, women without recorded CIN3+ during follow-up were classified as not having reached the CIN3+ endpoint.

HPV DNA status was defined by the cobas^®^ 4800 HPV screening result. Overall 7-type HPV mRNA positivity was defined as detection of E6/E7 mRNA from at least one of the seven genotypes included in PreTect HPV-Proofer’7: HPV16, 18, 31, 33, 45, 52, or 58. Women without detectable E6/E7 mRNA from any of these genotypes were classified as mRNA-negative.

For genotype-specific analyses, each mRNA-positive woman was assigned to one mutually exclusive index genotype. In women with more than one detected mRNA genotype, the index genotype was assigned according to a predefined hierarchy: HPV16 > HPV18 > HPV45 > HPV33 > HPV31 > HPV52 > HPV58. This attribution rule was used to avoid counting the same woman more than once in genotype-specific risk estimates and to maintain consistency with the Bodø comparison cohort. Co-infections, defined as detection of two or more mRNA genotypes in the same sample, were summarised separately.

### 2.5. HPV16/18-Specific and Supportive Analyses

Supportive analyses were performed to assess whether HPV mRNA testing further refined risk among women positive for HPV16 and/or HPV18 DNA, a subgroup commonly prioritised for intensified management in genotype-based screening algorithms. HPV16/18 DNA positivity was defined as detection of HPV16 and/or HPV18 by the cobas^®^ 4800 assay. HPV16/18 mRNA positivity was defined as detection of E6/E7 mRNA from HPV16 and/or HPV18 by PreTect HPV-Proofer’7.

Age-specific HPV16/18 DNA and mRNA positivity were summarised using predefined age groups: <25, 25–33, 34–69, and >69 years. These categories reflected the age structure of the Norwegian screening programme during the study period and allowed for comparison between younger women newly included in HPV-based screening and older women who had been eligible for HPV-based screening throughout most or all of the study period.

Within the HPV16/18 DNA-positive subgroup, CIN2+ risk was compared according to HPV16/18 mRNA status. This analysis was used to evaluate whether detection of E6/E7 mRNA could distinguish transcriptionally active HPV16/18 infections from HPV16/18 DNA positivity alone. Performance was summarised using sensitivity, specificity, positive predictive value, negative predictive value, and relative risk for CIN2+.

A separate referral simulation was performed within the HPV16/18 DNA-positive subgroup. Referral of all HPV16/18 DNA-positive women was compared with referral restricted to women who were also HPV16/18 mRNA-positive. Referral efficiency was expressed as the number of colposcopies required per detected CIN2+. This analysis was included to evaluate whether HPV16/18 mRNA testing could improve the targeting of colposcopy within a genotype group commonly prioritised in cervical screening algorithms.

### 2.6. Simulated Referral Strategies

To estimate the potential clinical impact of 7-type HPV mRNA triage, we performed a referral simulation within the HPV DNA-positive ASC-US/LSIL cohort. The simulation was based on observed test results and recorded CIN2+/CIN3+ outcomes but did not influence clinical management during the study period.

Two referral strategies were compared. The first assumed immediate colposcopy referral for all HPV DNA-positive women with ASC-US or LSIL cytology, representing the maximum referral scenario within the low-grade cytology subgroup. The second assumed immediate referral only for women with a positive 7-type HPV mRNA result, while women with a negative mRNA result would remain in structured surveillance with repeat HPV-based testing according to routine follow-up practice.

For each strategy, we calculated the number and proportion of women classified for immediate referral, the number of recorded CIN2+ and CIN3+ outcomes among these women, and, assuming one colposcopy per woman classified for immediate referral, the number of colposcopies per recorded CIN2+ or CIN3+ outcome. Referral efficiency was expressed as colposcopies per recorded CIN2+ outcome and colposcopies per recorded CIN3+ outcome.

The simulation also quantified CIN2+ and CIN3+ cases observed among mRNA-negative women. These cases represent lesions that would not be detected by immediate mRNA-guided referral alone and illustrate the need for continued surveillance after a negative mRNA triage result. The purpose of the simulation was to compare the reduction in immediate referral burden with the corresponding number of recorded high-grade outcomes among women classified for immediate referral, and to assess whether mRNA triage concentrated colposcopy referrals among women with higher observed risk during follow-up.

The simulated referral analysis represents a deterministic scenario based on observed test results and recorded CIN2+/CIN3+ outcomes. It assumes complete adherence to referral recommendations and one colposcopy per woman classified for immediate referral. Real-world implementation may differ because of variation in clinical decision-making, follow-up adherence, colposcopy practice, and local programme algorithms.

### 2.7. Statistical Analysis

Descriptive statistics were used to characterise the Tromsø ASC-US/LSIL cohort and to summarise HPV DNA, cytology, HPV mRNA, genotype, histological follow-up, and outcome distributions. Results are presented as counts and percentages. CIN2+ and CIN3+ risks were calculated as the proportion of women with the respective recorded endpoint within each cytology, mRNA, genotype, or referral-defined subgroup.

Diagnostic performance of overall 7-type HPV mRNA triage was evaluated using 2 × 2 contingency tables. Sensitivity, specificity, positive predictive value (PPV), and negative predictive value (NPV) were calculated separately for CIN2+ and CIN3+. Relative risks (RRs) were used to compare recorded CIN2+ and CIN3+ risks between mRNA-positive and mRNA-negative women. Differences in proportions within the Tromsø cohort were assessed using Pearson’s χ^2^ test, with Fisher’s exact test being used when expected cell counts were small. Diagnostic performance estimates in the full cohort were calculated against recorded CIN2+ and CIN3+ endpoints during routine follow-up and should not be interpreted as accuracy estimates against a uniformly applied histological reference standard.

Genotype-specific risk was evaluated using mutually exclusive index-genotype attribution among mRNA-positive women. Genotype-specific PPVs for CIN2+ and CIN3+ were calculated for each index genotype. Co-infections, defined as detection of two or more mRNA genotypes in the same sample, were summarised descriptively but were not analysed as separate risk categories because of the small numbers.

Supportive analyses focused on HPV16/18. HPV16/18 DNA and HPV16/18 mRNA positivity were summarised by age group, and CIN2+ risk was compared between HPV16/18 mRNA-positive and HPV16/18 mRNA-negative women within the HPV16/18 DNA-positive subgroup.

Histological follow-up status and the most severe recorded histological diagnosis were summarised overall and stratified by 7-type HPV mRNA status. Because histological verification was determined by routine clinical management and was not systematic, no separate diagnostic-accuracy analysis was performed within the histologically verified subgroup.

Referral efficiency was evaluated using deterministic simulations based on observed cohort data. Two strategies were compared: immediate referral of all HPV DNA-positive women with ASC-US/LSIL cytology and immediate referral restricted to women with a positive 7-type HPV mRNA result. For each strategy, we calculated the number and proportion of women classified for immediate referral, the number of recorded CIN2+ and CIN3+ outcomes among these women, and, assuming one colposcopy per woman classified for immediate referral, the number of colposcopies per recorded CIN2+ or CIN3+ outcome.

Selected Tromsø findings were compared descriptively with the previously published Bodø cohort to describe cross-cohort patterns and explore potential interlaboratory differences in low-grade cytology classification. The comparison included the proportion of HPV DNA-positive women classified as ASC-US/LSIL, 7-type HPV mRNA positivity, recorded CIN2+ and CIN3+ risks according to mRNA status, and simulated referral reduction. No formal statistical testing was performed for the Tromsø–Bodø comparison because the cohorts differed in study period, population size, inclusion procedures, and follow-up definitions.

All analyses were performed using IBM SPSS Statistics for Windows, version 29.0. A two-sided *p*-value < 0.05 was considered statistically significant for analyses within the Tromsø cohort. The referral simulations were deterministic and intended to describe referral burden and diagnostic yield under predefined scenarios rather than to estimate the causal effect of a clinical intervention. The Tromsø–Bodø comparison was descriptive and exploratory.

### 2.8. Ethics

This retrospective register-based quality-assurance study was approved by the Regional Committee for Medical and Health Research Ethics, North Norway (REK Nord 203384). The study was based on routinely recorded data from the laboratory information system SymPathy at the Department of Clinical Pathology, University Hospital of North Norway. No patients were contacted, no additional samples were collected, and clinical follow-up was not altered as a consequence of the study. All analyses were performed on a de-identified dataset. In accordance with Norwegian regulations for quality-assurance studies using de-identified registry data, individual informed consent was not required. The study was conducted in accordance with institutional and national regulations and the principles of the Declaration of Helsinki.

## 3. Results

### 3.1. Study Population

From January 2019 through December 2024, 42,791 women underwent primary HPV DNA screening at the University Hospital of North Norway. HPV DNA was detected in 2405 women (5.6%), while 40,386 women (94.4%) tested HPV DNA-negative. Among HPV DNA-positive women, 37 were excluded from the linked analysis: 31 because of insufficient residual sample volume, four because of an invalid 7-type HPV mRNA result, and two because a Norwegian personal identification number was unavailable for reliable linkage. The linked HPV DNA-positive cohort therefore comprised 2368 women.

**Figure 1:**
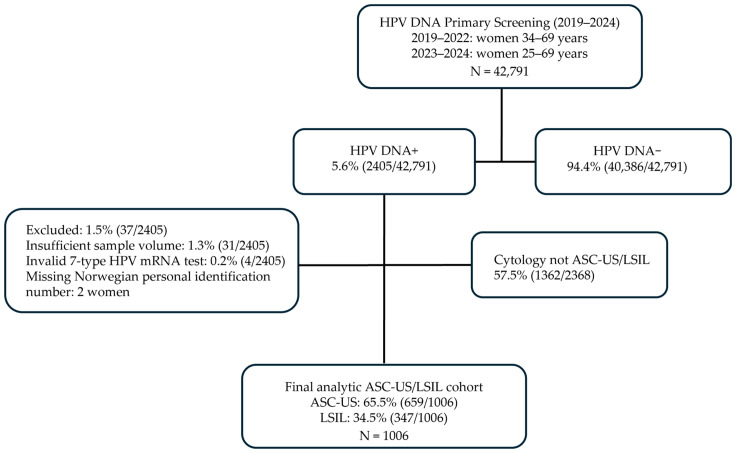
Flowchart of the study population. From January 2019 through December 2024, 42,791 women underwent primary HPV DNA screening at the University Hospital of North Norway. Of these, 2405 women (5.6%) tested positive for HPV DNA and 40,386 (94.4%) tested negative. Among HPV DNA-positive women, 37 were excluded from the linked analysis: 31 because of insufficient residual sample volume, four because of an invalid 7-type HPV mRNA result, and two because a Norwegian personal identification number was unavailable for reliable linkage. The linked HPV DNA-positive cohort comprised 2368 women. Among these, 1362 women (57.5%) had reflex cytology other than ASC-US or LSIL and were not included in the present analysis. The final analytic cohort comprised 1006 HPV DNA-positive women with ASC-US/LSIL cytology, including 659 women with ASC-US (65.5%) and 347 women with LSIL (34.5%). Abbreviations: HPV, human papillomavirus; DNA, deoxyribonucleic acid; mRNA, messenger ribonucleic acid; ASC-US, atypical squamous cells of undetermined significance; LSIL, low-grade squamous intraepithelial lesion.

Among these 2368 women, 1362 (57.5%) had reflex cytology other than ASC-US or LSIL and were not included in the present analysis. The final analytic cohort comprised 1006 HPV DNA-positive women with ASC-US/LSIL cytology, including 659 women with ASC-US (65.5%) and 347 women with LSIL (34.5%) (Figure 1). All included women had valid reflex cytology and 7-type HPV E6/E7 mRNA results, with linkage to follow-up pathology records through 31 December 2025.

The 7-type HPV mRNA assay was positive in 410 of 1006 women (40.8%) and negative in 596 of 1006 women (59.2%). mRNA positivity was similar in the ASC-US and LSIL subgroups: 273 of 659 women with ASC-US (41.4%) and 137 of 347 women with LSIL (39.5%; χ^2^ = 0.36, *p* = 0.551).

CIN2+ was recorded in 154 of 1006 women (15.3%), and CIN3+ in 22 of 1006 women (2.2%). CIN2+ was more frequent among women with LSIL than among women with ASC-US cytology (65/347, 18.7% vs. 89/659, 13.5%). In contrast, CIN3+ detection was similar in the ASC-US and LSIL subgroups (15/659, 2.3% vs. 7/347, 2.0%) (Table 1).

### 3.2. Histological Follow-Up and Verification Status

Relevant histology was recorded in 537 of 1006 women (53.4%) and was more frequent among mRNA-positive than mRNA-negative women (63.7% vs. 46.3%; Table 2). A recorded CIN2+ endpoint was identified in 154 women, comprising 132 women with CIN2, 21 with CIN3, and one with squamous cell carcinoma. Because histological verification was performed according to routine clinical management and was not systematic, women without recorded CIN2+ or CIN3+ were classified as having no recorded endpoint during the available follow-up period, rather than as having histologically confirmed absence of disease.

### 3.3. Diagnostic Performance of mRNA Triage

The diagnostic performance of 7-type HPV mRNA triage was evaluated by comparing women with detectable E6/E7 mRNA from at least one of the seven targeted genotypes (HPV16, 18, 31, 33, 45, 52, or 58) with women who were mRNA-negative.

For CIN2+, 107 of 410 mRNA-positive women had recorded CIN2+, corresponding to a risk of 26.1%. In comparison, CIN2+ was recorded in 47 of 596 mRNA-negative women, corresponding to a risk of 7.9%. Thus, mRNA-positive women had a more than threefold higher CIN2+ risk than mRNA-negative women (RR 3.31, 95% CI 2.41–4.55; *p* < 0.001). The sensitivity of 7-type HPV mRNA triage for CIN2+ was 69.5%, with a specificity of 64.4%, PPV of 26.1%, and NPV of 92.1% (Table 3A). Overall, 47 of 154 CIN2+ cases (30.5%) occurred among mRNA-negative women and would not have been referred at baseline using immediate mRNA-guided referral alone.

For CIN3+, 16 of 410 mRNA-positive women had recorded CIN3+, corresponding to a risk of 3.9%, compared with 6 of 596 mRNA-negative women (1.0%). The relative risk of CIN3+ was therefore higher among mRNA-positive women (RR 3.88, 95% CI 1.53–9.82; *p* = 0.002). The corresponding test characteristics for CIN3+ were sensitivity 72.7%, specificity 60.0%, PPV 3.9%, and NPV 99.0% (Table 3B). Six of 22 CIN3+ cases (27.3%) occurred among mRNA-negative women, indicating that mRNA-negative women still require structured follow-up, although their absolute CIN3+ risk was low.

### 3.4. Genotype-Specific Risk Stratification

Genotype-specific CIN2+ and CIN3+ risks are summarised in Table 4. The highest observed CIN2+ PPVs were associated with HPV16 and HPV18 mRNA, followed by HPV31 and HPV33. For CIN3+, the highest observed PPVs were associated with HPV16 and HPV33. Estimates for several genotypes were based on small numbers and should be interpreted descriptively rather than as definitive genotype rankings.

### 3.5. Co-Infections Detected by the 7-Type HPV mRNA Assay

Co-infections with two or more mRNA genotypes were detected in 26 of 410 mRNA-positive women (6.3%), corresponding to 2.6% of the full analytic cohort. HPV33 + HPV58 was the most frequent pattern and was observed in six women; all other co-infection patterns occurred in one or two women each (Table 5). Because individual patterns were uncommon, they were not analysed as separate risk categories.

### 3.6. HPV16/18 DNA–mRNA Discordance and CIN2+ Risk Stratification

HPV16/18 DNA was detected in 184 of 1006 women (18.3%), whereas HPV16/18 E6/E7 mRNA was detected in 117 women (11.6%), corresponding to a 36.4% lower positivity rate with mRNA detection. The DNA–mRNA difference was greater among women aged 34–69 years than among women aged 25–33 years (Table 6A).

Within the HPV16/18 DNA-positive subgroup, CIN2+ was recorded in 42.7% of HPV16/18 mRNA-positive women and 13.4% of HPV16/18 mRNA-negative women. Diagnostic performance and referral efficiency are summarised in Table 6B. Restricting simulated immediate referral to HPV16/18 mRNA-positive women reduced the number of colposcopies per recorded CIN2+ from 3.12 to 2.34.

### 3.7. Simulated Referral Strategies and Procedural Yield

Under the simulated mRNA-guided strategy, immediate referrals decreased from 1006 to 410 women, corresponding to a 59.2% reduction. The number of colposcopies per recorded CIN2+ decreased from 6.5 to 3.8, and the number per recorded CIN3+ decreased from 45.7 to 25.6. However, 47 CIN2+ cases and 6 CIN3+ cases occurred among mRNA-negative women and would depend on structured surveillance for detection (Table 7).

### 3.8. Descriptive Comparison with the Bodø Cohort

ASC-US/LSIL accounted for 42.5% of linked HPV DNA-positive women in Tromsø and 22.3% in Bodø. Despite this difference in cytology distribution, 7-type mRNA positivity was similar in the two analytic cohorts, at 40.8% and 44.6%, respectively.

In both cohorts, recorded CIN2+ and CIN3+ risks were higher among mRNA-positive than mRNA-negative women, although absolute risks differed between settings. The simulated reduction in immediate referrals was 59.2% in Tromsø and 55% in Bodø. Because the cohorts differed in study period, size, inclusion procedures, and follow-up definitions, the comparison was descriptive and no formal statistical testing was performed (Table 8).

## 4. Discussion

### 4.1. Principal Findings and Descriptive Comparison with the Bodø Cohort

In this population-based cohort of HPV DNA-positive women with ASC-US/LSIL cytology, genotype-specific 7-type HPV E6/E7 mRNA testing separated women into groups with different recorded risks of high-grade cervical disease. CIN2+ was recorded in 26.1% of mRNA-positive women and 7.9% of mRNA-negative women, whereas the corresponding CIN3+ risks were 3.9% and 1.0%. Thus, mRNA positivity was associated with clear risk enrichment, although the absolute CIN3+ risk remained low in both groups. The clinical relevance of the assay is therefore best understood as risk stratification rather than binary exclusion of disease.

The simulated referral analysis indicated that restricting immediate referral to mRNA-positive women could reduce the number of immediate colposcopy referrals by 59.2% and concentrate referrals among women with a higher recorded disease yield. However, 47 of 154 CIN2+ cases and 6 of 22 CIN3+ cases occurred among mRNA-negative women. Accordingly, mRNA triage alone cannot safely exclude clinically relevant disease and should not be used to return mRNA-negative women directly to routine screening. Any implementation would require a predefined surveillance pathway, including repeat HPV-based testing and referral according to persistent infection, repeat triage results, cytological progression, or other risk indicators [3,16,17,21].

The Tromsø cohort also provides a larger population-based evaluation of the same genotype-specific assay previously studied in Bodø [29]. The proportion of HPV DNA-positive women classified as having ASC-US/LSIL was markedly higher in Tromsø than in Bodø. This difference may reflect variation in cytological thresholds, laboratory practice, population characteristics, screening implementation, study period, or follow-up definitions. Observer and interlaboratory variability are well recognised for borderline and low-grade cytology categories and can influence both referral volume and the risk composition of cytology-defined subgroups [10,13,15].

Despite these differences, both cohorts showed directionally similar patterns: mRNA-positive women had higher recorded disease risks than mRNA-negative women, and simulated mRNA-guided triage reduced immediate referral volume [29]. However, the Tromsø–Bodø comparison was descriptive, and no formal between-cohort statistical testing was performed. The findings should therefore not be interpreted as formal external validation or evidence of assay reproducibility. Rather, they suggest that similar patterns of molecular risk stratification may be observed in two routine laboratory settings despite substantial differences in the proportion of HPV DNA-positive women assigned to ASC-US/LSIL cytology.

The principal contribution of the present study is therefore not replication alone. It provides risk estimates from a substantially larger population-based ASC-US/LSIL cohort, evaluates genotype-specific and HPV16/18-specific risk refinement, and illustrates how variation in cytological classification may influence the size and apparent disease prevalence of a low-grade triage population. The findings support further evaluation of genotype-specific mRNA testing as an objective molecular component of risk-based triage, while also demonstrating that structured surveillance remains necessary after a negative result.

### 4.2. Genotype-Specific and HPV16/18 Risk Refinement

HPV DNA testing identifies the presence and genotype of viral DNA but does not establish whether the detected infection has measurable viral oncogene transcription. In contrast, E6/E7 mRNA detection provides evidence of transcriptional activity involving viral oncogenes that contribute directly to HPV-mediated cellular transformation [21,22]. Genotype-specific mRNA testing therefore combines two distinct sources of risk information: the HPV genotype detected and the presence of E6/E7 transcription from that genotype.

The genotype-specific results indicate that mRNA-positive women do not constitute a uniform risk group. The highest recorded CIN2+ risks were observed among women assigned to HPV16, HPV18, HPV31, or HPV33 mRNA, whereas CIN3+ was most clearly enriched among women assigned to HPV16 or HPV33. These findings are broadly consistent with established differences in carcinogenic risk between HPV genotypes [21,24]. However, several genotype strata were small, and the estimates should be interpreted descriptively rather than as definitive rankings. In particular, no CIN3+ cases were recorded among women assigned to HPV18 mRNA, but this finding does not imply absence of biological or clinical risk and likely reflects the limited size of the HPV18 stratum and the available follow-up.

The HPV16/18 analysis further showed that women positive for HPV16 and/or HPV18 DNA did not form a uniform risk group. Detection of HPV16/18 E6/E7 mRNA identified a subgroup with a higher recorded CIN2+ risk than HPV16/18 DNA-positive/mRNA-negative women. This suggests that mRNA testing may add risk information within genotype groups that are already prioritised in screening algorithms [21]. Nevertheless, CIN2+ was also recorded among HPV16/18 DNA-positive/mRNA-negative women. HPV16/18 mRNA negativity should therefore not be interpreted as absence of risk or as justification for withdrawal from follow-up.

The observed difference between HPV16/18 DNA and mRNA positivity was larger among women aged 34–69 years than among women aged 25–33 years. This may reflect age-related differences in infection duration, viral activity, previous screening, or cohort composition. However, the study was not designed to test age-specific biological mechanisms, and the age groups were unevenly represented. The age-specific findings should therefore be regarded as exploratory.

Earlier studies of HPV E6/E7 mRNA testing have generally reported lower test positivity and greater specificity than broader HPV DNA-based strategies, although assay design, genotype coverage, study population, and clinical endpoint differ substantially between studies [22,23,26,27,28]. Studies of the 7-type assay in broader HPV DNA-positive populations have similarly reported improved referral efficiency relative to cytology- or genotype-based strategies, accompanied by some reduction in sensitivity [23,30,31]. International studies have also supported an association between HPV mRNA positivity and increased risk of high-grade disease in HPV-positive or cytologically abnormal populations [28,32,33,34]. The present findings extend this evidence within a large, routinely screened ASC-US/LSIL cohort, while reinforcing that the clinical value of mRNA testing depends on how positive and negative results are incorporated into a complete risk-based management pathway.

### 4.3. Clinical and Programmatic Implications

The central clinical challenge after a positive HPV DNA screening result is to identify women who require prompt diagnostic evaluation while avoiding unnecessary procedures among women with transient infection or lower immediate risk. Available triage and management approaches include reflex cytology, partial or extended HPV genotyping, p16/Ki-67 dual-stain cytology, methylation-based biomarkers, repeat HPV testing, and, in some settings, colposcopy-based triage [4,5,8,21,35]. These approaches differ in their biological basis, specimen requirements, diagnostic role, and implications for referral and follow-up, and may be used alone or in combination.

Cytology provides morphological information and remains valuable for identifying high-grade cellular abnormalities, but is observer-dependent and requires a suitable cervical cytology specimen [8,9,10,13]. Extended HPV genotyping provides type-specific risk information but measures viral presence rather than oncogene transcription [21,22,24]. Dual-stain cytology identifies host–cell protein expression associated with transforming infection, whereas methylation assays detect epigenetic changes associated with disease progression [4,5,8]. Genotype-specific E6/E7 mRNA testing addresses a complementary biological question by detecting viral oncogene transcription from selected genotypes [22,23].

The present study did not directly compare mRNA testing with dual-stain cytology, methylation-based triage, repeat HPV testing, or alternative extended-genotyping algorithms and therefore cannot establish superiority over these approaches. Direct head-to-head studies using the same population, reference standard, follow-up period, and management thresholds are needed to compare sensitivity, specificity, referral burden, safety, and programme-level efficiency.

The simulated reduction in immediate referrals suggests that mRNA triage could reduce colposcopy workload, use of specialist services, and the number of women undergoing immediate diagnostic procedures. This may reduce patient burden associated with additional appointments, travel, anxiety and invasive assessment. However, under an mRNA-guided strategy, mRNA-negative women would require structured surveillance, repeat testing, and possible later referral. Fewer immediate referrals may therefore shift rather than eliminate healthcare-resource use.

The number of colposcopies per recorded CIN2+ or CIN3+ outcome is a measure of procedural efficiency and should not be interpreted as an estimate of cost per case detected. The present study did not include assay and laboratory costs, staff time, surveillance visits, repeat HPV or triage testing, patient travel, loss to follow-up, later colposcopies, biopsy or treatment costs, or costs per detected CIN2+ or CIN3+. Referral reduction alone therefore does not establish lower total programme costs or cost-effectiveness. Formal health-economic analyses should compare total pathway costs, healthcare-resource utilisation, cost per detected CIN2+ and CIN3+, and clinical outcomes for mRNA-guided triage versus alternative triage strategies [16,17].

The referral analysis was deterministic and assumed complete adherence to recommendations—one colposcopy per woman classified for immediate referral, and continued structured follow-up of mRNA-negative women. Real-world outcomes may differ because of variation in clinician decisions, patient adherence, laboratory and colposcopy capacity, repeat-testing practices, and local programme algorithms. Consequently, the estimated 59.2% reduction should be interpreted as a modelled reduction in immediate referrals within this cohort rather than as evidence of an equivalent reduction in total resource use or programme costs, or as a directly transportable effect in other screening programmes.

Molecular triage may become increasingly relevant as HPV self-sampling expands. Self-collected samples are suitable for molecular HPV testing but generally do not provide a conventional clinician-collected cervical specimen for reflex cytology or cytology-based dual staining [4,8,18,19,20]. Cytology-dependent triage may therefore require recall for an additional sample, creating an additional visit and a potential point of loss to follow-up. Molecular testing that can be performed on the same or a compatible self-collected specimen could simplify this pathway.

Previous work has demonstrated the feasibility of 7-type HPV E6/E7 mRNA testing in self-collected material [36]. However, the present study used clinician-collected PreservCyt samples and does not establish clinical performance in primary self-sampling programmes. Performance may depend on the collection device, cellular yield, transport conditions, sample medium, pre-analytical processing, and laboratory workflow. Further validation is therefore required before the present findings can be extrapolated to home-based self-sampling or other collection systems.

Overall, genotype-specific mRNA testing may have a role as an additional molecular risk-stratification step after HPV DNA positivity, either as a complement to cytology and genotyping or, in selected workflows, as an alternative molecular triage approach. Its clinical use should be embedded within an organised programme that defines which women are referred immediately, how mRNA-negative women are followed, and how persistent HPV positivity or changing risk indicators trigger further investigation.

### 4.4. Strengths, Limitations, and Future Research

A major strength of this study is the large, population-based cohort derived from routine primary HPV screening. The analysis included 1006 HPV DNA-positive women with ASC-US/LSIL cytology and used individual-level linkage to regional pathology records. Because the University Hospital of North Norway is the sole pathology provider for Troms and Finnmark, the dataset provides broad regional capture of HPV testing, cytology, biopsy, conisation, and other relevant pathology records. HPV DNA testing, reflex cytology, and mRNA testing were performed using material from the same screening episode, and the retrospective mRNA results did not influence referral, biopsy, treatment, or follow-up.

Several limitations require careful consideration. Histological verification was performed according to routine clinical management and was not systematic for all women. Women without recorded CIN2+ or CIN3+ were classified as having no recorded endpoint during the available observation period rather than as having a histologically confirmed negative outcome. Consequently, disease may have been under-ascertained among women managed by surveillance without biopsy, women with lesions not detected during routine assessment, and women with shorter available follow-up.

This limitation may affect estimates of recorded risk, sensitivity, specificity, and negative predictive value. Although the retrospective mRNA result did not influence biopsy decisions, histological verification depended on routine HPV DNA- and cytology-based management and may therefore have differed according to baseline clinical risk. This introduces the possibility of partial verification bias. To improve transparency, we reported the proportion of women with relevant histology and the most severe recorded histological diagnosis, stratified by mRNA status. Cytology- and/or HPV-based follow-up without histology was not separately classified in the present data extraction and could therefore not be distinguished from absence of later registered testing. Because verification was clinically selected rather than systematic, the reported histological distributions do not represent an unbiased diagnostic-accuracy analysis.

Follow-up duration also varied because women entered the cohort between 2019 and 2024, whereas pathology records were reviewed through 31 December 2025. Women screened in later years had less time for CIN2+ or CIN3+ to be diagnosed. This is particularly relevant for CIN3+, which may develop or be detected over a longer interval. The limited number of recorded CIN3+ cases reduced precision, especially in genotype-specific analyses, and longer follow-up is required to assess cumulative risk after a negative 7-type mRNA result.

The study population was deliberately restricted to HPV DNA-positive women with ASC-US/LSIL cytology. Absolute risks, predictive values, and simulated referral estimates therefore apply to an already risk-enriched triage population and should not be extrapolated directly to an unselected screening population or to HPV DNA-positive women with other cytology results. The assay detects E6/E7 mRNA from seven genotypes; accordingly, the mRNA-negative group includes women with HPV DNA positivity for non-targeted genotypes as well as women without detectable transcription from the targeted genotypes. An mRNA-negative result should therefore be interpreted as absence of detectable E6/E7 mRNA from the seven assay targets, not as absence of HPV infection or disease risk.

The study was conducted within a single organised Norwegian healthcare system with extensive pathology linkage and established capacity for longitudinal follow-up. The findings may not be directly transferable to settings with different HPV prevalence, vaccination coverage, screening participation, laboratory infrastructure, colposcopy capacity, or follow-up adherence. Information on vaccination status, smoking, immunosuppression, and other individual risk modifiers was not available, limiting adjustment for factors that may influence HPV persistence and disease progression.

Future research should prioritise prospective multicentre evaluation with standardised follow-up and clearly defined management algorithms. Such studies should report cumulative CIN2+ and CIN3+ risks, particularly among mRNA-negative women, and should assess how repeat HPV testing, genotype persistence, repeat mRNA testing, cytology, and screening history can be combined to define safe surveillance intervals. Direct comparisons with extended genotyping, dual-stain cytology, methylation-based triage, repeat HPV testing, and colposcopy-based strategies are needed within the same populations.

Programme-level implementation studies should additionally evaluate adherence, colposcopy use, patient burden, laboratory workflow, and cost-effectiveness. Validation in increasingly vaccinated cohorts and in specific self-sampling systems will also be important because HPV prevalence, genotype distribution, specimen characteristics, and absolute disease risks are likely to differ from those observed in the present cohort.

## 5. Conclusions

In this population-based cohort of HPV DNA-positive women with ASC-US/LSIL cytology, genotype-specific 7-type HPV E6/E7 mRNA testing identified a subgroup with higher recorded risks of CIN2+ and CIN3+. HPV16/18 mRNA testing also provided additional risk stratification among women already positive for HPV16/18 DNA, indicating that detection of viral oncogene transcription may add information beyond HPV DNA genotype alone.

In simulated referral analyses, mRNA-guided triage reduced immediate colposcopy referrals by 59.2% and improved referral efficiency for both CIN2+ and CIN3+. These findings indicate that genotype-specific mRNA testing may help concentrate immediate referral among women with higher recorded disease risk and reduce unnecessary diagnostic procedures.

However, clinically relevant CIN2+ and CIN3+ outcomes were also recorded among mRNA-negative women. The assay should therefore be regarded as a risk-stratification tool rather than a stand-alone rule-out test, and mRNA-negative women should remain in a predefined structured surveillance pathway.

The Tromsø and Bodø cohorts showed directionally similar patterns of risk stratification despite differences in cohort size, study period, cytology distribution, and follow-up. Because the comparison was descriptive, the findings should not be interpreted as formal external validation. Prospective evaluation with standardised follow-up, direct comparison with alternative triage methods, and health-economic assessment are needed before programme-level implementation.

## Figures and Tables

**Table 1:** Cohort characteristics, cytology distribution, mRNA positivity, and recorded CIN2+/CIN3+ endpoints (*N* = 1006).

Characteristic	*n/N* (*%*)
Cytology	
ASC-US	659/1006 (65.5)
LSIL	347/1006 (34.5)
Recorded endpoints during follow-up	
Recorded CIN2+ overall	154/1006 (15.3)
ASC-US subgroup	89/659 (13.5)
LSIL subgroup	65/347 (18.7)
Recorded CIN3+ overall	22/1006 (2.2)
ASC-US subgroup	15/659 (2.3)
LSIL subgroup	7/347 (2.0)
mRNA (7-type) positive	410/1006 (40.8)
ASC-US subgroup	273/659 (41.4)
LSIL subgroup	137/347 (39.5)

Abbreviations: ASC-US, atypical squamous cells of undetermined significance; LSIL, low-grade squamous intraepithelial lesion; CIN, cervical intraepithelial neoplasia; mRNA, messenger ribonucleic acid.

**Table 2:** Histological follow-up status through 31 December 2025, overall and according to 7-type HPV mRNA status.

Follow-Up Category	Total (*N* = 1006), *n*/*N* (%)	mRNA Positive (*n* = 410), *n*/*N* (%)	mRNA Negative (*n* = 596), *n*/*N* (%)
No relevant histology recorded	469/1006 (46.6)	149/410 (36.3)	320/596 (53.7)
Any histological follow-up	537/1006 (53.4)	261/410 (63.7)	276/596 (46.3)
Normal	63/1006 (6.3)	19/410 (4.6)	44/596 (7.4)
Unsatisfactory	1/1006 (0.1)	1/410 (0.2)	0/596 (0.0)
CIN1	319/1006 (31.7)	134/410 (32.7)	185/596 (31.0)
CIN2	132/1006 (13.1)	91/410 (22.2)	41/596 (6.9)
CIN3	21/1006 (2.1)	16/410 (3.9)	5/596 (0.8)
ACIS	0/1006 (0.0)	0/410 (0.0)	0/596 (0.0)
SCC/ACC	1/1006 (0.1)	0/410 (0.0)	1/596 (0.2)
Total	1006/1006 (100.0)	410/410 (100.0)	596/596 (100.0)

Note: Percentages are calculated within each column. Histological follow-up was categorised according to the most severe relevant histological diagnosis recorded on or after the index screening date through 31 December 2025. Cytology/HPV-only follow-up was not included in the present histological follow-up classification. SCC/ACC includes one recorded SCC case and no recorded ACC cases; no ACIS cases were recorded. Abbreviations: ACIS, adenocarcinoma in situ; ACC, adenocarcinoma; CIN, cervical intraepithelial neoplasia; HPV, human papillomavirus; mRNA, messenger ribonucleic acid; SCC, squamous cell carcinoma.

**Table 3:** Diagnostic performance of 7-type HPV mRNA triage among HPV DNA-positive women with ASC-US/LSIL cytology (*N* = 1006). (**A**) CIN2+ endpoint. (**B**) CIN3+ endpoint.

(**A**)				
**mRNA Result**	**CIN2+ (** ***n*** **)**	**No Recorded CIN2+ (** ***n*** **)**	**Total (** ***n*** **)**	**Predictive Value**
Positive	107	303	410	PPV 26.1%
Negative	47	549	596	NPV 92.1%
Total	154	852	1006	-
(**B**)				
**mRNA Result**	**CIN3+ (** ***n*** **)**	**No Recorded CIN3+ (** ***n*** **)**	**Total (** ***n*** **)**	**Predictive Value**
Positive	16	394	410	PPV 3.9%
Negative	6	590	596	NPV 99.0%
Total	22	984	1006	-

Abbreviations: ASC-US, atypical squamous cells of undetermined significance; CIN, cervical intraepithelial neoplasia; DNA, deoxyribonucleic acid; HPV, human papillomavirus; LSIL, low-grade squamous intraepithelial lesion; mRNA, messenger ribonucleic acid; NPV, negative predictive value; PPV, positive predictive value; RR, relative risk.

**Table 4:** Genotype-specific positive predictive values (PPVs) for CIN2+ and CIN3+ with 7-type HPV mRNA triage.

Index mRNA Genotype	*N* Assigned	CIN2+ *n* (PPV%)	CIN3+ *n* (PPV%)
16	87	39 (44.8%)	9 (10.3%)
18	30	11 (36.7%)	0 (0.0%)
31	83	21 (25.3%)	2 (2.4%)
33	35	9 (25.7%)	3 (8.6%)
45	72	10 (13.9%)	0 (0.0%)
52	79	15 (19.0%)	2 (2.5%)
58	24	2 (8.3%)	0 (0.0%)
Total	410	107 (26.1%)	16 (3.9%)

Note: Genotype-specific PPVs were calculated using hierarchical index genotype assignment for women with multiple detected mRNA genotypes. The total row represents all mRNA-positive women included in the genotype-specific analysis.

**Table 5:** Distribution of co-infections detected by the 7-type HPV E6/E7 mRNA assay among mRNA-positive women (*n* = 410).

mRNA Genotypes Detected	*n/N* (%)
HPV33 + HPV58	6/410 (1.5)
HPV16 + HPV52	2/410 (0.5)
HPV16 + HPV58	2/410 (0.5)
HPV31 + HPV52	2/410 (0.5)
HPV33 + HPV45	2/410 (0.5)
HPV45 + HPV52	2/410 (0.5)
HPV16 + HPV18	1/410 (0.2)
HPV16 + HPV31 + HPV52	1/410 (0.2)
HPV16 + HPV33	1/410 (0.2)
HPV16 + HPV52 + HPV58	1/410 (0.2)
HPV18 + HPV31	1/410 (0.2)
HPV31 + HPV33 + HPV58	1/410 (0.2)
HPV31 + HPV45	1/410 (0.2)
HPV31 + HPV58	1/410 (0.2)
HPV33 + HPV45 + HPV58	1/410 (0.2)
HPV45 + HPV58	1/410 (0.2)

Note: Co-infection was defined as detection of two or more mRNA genotypes in the same sample. Individual patterns were not analysed as separate risk categories because of the small counts.

**Table 6:** HPV16/18 DNA–mRNA discordance and CIN2+ risk stratification.

(**A**) **Age-Specific HPV16/18 Positivity**				
**Age Group,** **Years**	***N***	**HPV16/18 DNA-Positive,** ***n/N*** **(%)**	**HPV16/18 mRNA-Positive,** ***n/N*** **(%)**	**Relative Reduction**
25–33	142	26/142 (18.3)	23/142 (16.2)	11.5%
34–69	859	158/859 (18.4)	94/859 (10.9)	40.5%
Total	1006	184/1006 (18.3)	117/1006 (11.6)	36.4%
(**B**) **CIN2+ Risk Among HPV16/18 DNA-Positive Women**				
**HPV16/18 Subgroup**	***N***	**Recorded CIN2+,** ***n/N*** **(%)**	**No Recorded CIN2+,** ***n/N*** **(%)**	**Colposcopies Per Recorded** **CIN2+**
All HPV16/18 DNA-positive	184	59/184 (32.1)	125/184 (67.9)	3.12
HPV16/18 mRNA-positive	117	50/117 (42.7)	67/117 (57.3)	2.34
HPV16/18 mRNA-negative	67	9/67 (13.4)	58/67 (86.6)	—

Note: (**A**) Five women were younger than 25 years, and none were HPV16/18 DNA- or mRNA-positive. No women were older than 69 years. Relative reduction was calculated as the proportional decrease in HPV16/18 positivity for mRNA compared with DNA detection. (**B**) For HPV16/18 mRNA triage of HPV16/18 DNA-positive women, sensitivity was 84.7%, specificity 46.4%, PPV 42.7%, NPV 86.6%, and RR 3.18.

**Table 7:** Simulated immediate referral strategies among HPV DNA-positive women with ASC-US/LSIL cytology.

Referral Strategy	Immediate Referrals, *n/N* (%)	CIN2+ Included, *n/N* (%)	Colposcopies Per CIN2+	CIN3+ Included, *n/N* (%)	Colposcopies Per CIN3+
Refer all women	1006/1006 (100.0)	154/154 (100.0)	6.5	22/22 (100.0)	45.7
Refer mRNA-positive women	410/1006 (40.8)	107/154 (69.5)	3.8	16/22 (72.7)	25.6

Note: The deterministic simulation assumed complete adherence and one colposcopy per woman classified for immediate referral. Under the mRNA-guided strategy, 47 of 154 CIN2+ cases and 6 of 22 CIN3+ cases occurred among women not classified for immediate referral and would depend on structured surveillance for detection.

**Table 8:** Descriptive comparison of the Tromsø and Bodø ASC-US/LSIL cohorts.

Characteristic	Tromsø	Bodø
HPV DNA-positive women available for comparison	2368	1243
ASC-US/LSIL among HPV DNA-positive women	1006/2368 (42.5%)	277/1243 (22.3%)
Analytic ASC-US/LSIL cohort	1006	175
7-type mRNA-positive	410/1006 (40.8%)	78/175 (44.6%)
Recorded CIN2+ overall	154/1006 (15.3%)	41/175 (23.4%)
Recorded CIN2+, mRNA-positive	107/410 (26.1%)	26/78 (33.3%)
Recorded CIN2+, mRNA-negative	47/596 (7.9%)	15/97 (15.5%)
Recorded CIN3+, mRNA-positive	16/410 (3.9%)	11/78 (14.1%)
Recorded CIN3+, mRNA-negative	6/596 (1.0%)	6/97 (6.2%)
Simulated referral reduction	59.2%	55%

Note: The Tromsø cohort covered screening during 2019–2024, whereas the Bodø cohort covered 2022–2024. The cohorts differed in size, inclusion procedures, screening implementation, and follow-up definitions. The comparison was descriptive, and no formal between-cohort statistical testing was performed.

## Data Availability

The individual-level data presented in this study are not publicly available due to privacy and ethical restrictions under Norwegian data protection regulations. De-identified aggregate cross-tabulated data supporting the main analyses, including triage results and CIN2+ and CIN3+ outcomes, may be made available from the corresponding author upon reasonable request.

## Abbreviations

The following abbreviations are used in this manuscript:

ASC-US	Atypical squamous cells of undetermined significance
CI	Confidence interval
CIN2+	Cervical intraepithelial neoplasia grade 2 or worse
CIN3+	Cervical intraepithelial neoplasia grade 3 or worse
DNA	Deoxyribonucleic acid
E6/E7	HPV E6/E7 viral oncogenes
HPV	Human papillomavirus
LSIL	Low-grade squamous intraepithelial lesion
mRNA	Messenger ribonucleic acid
NPV	Negative predictive value
PPV	Positive predictive value
RR	Relative risk
SPSS	Statistical Package for the Social Sciences
UNN	University Hospital of North Norway
